# Effects of Healthy Lifestyles on Chronic Diseases: Diet, Sleep and Exercise

**DOI:** 10.3390/nu15214627

**Published:** 2023-10-31

**Authors:** Henrik Oster, Inês Chaves

**Affiliations:** 1 Institute of Neurobiology, Center for Brain, Behavior & Metabolism (CBBM), University of Luebeck, 23562 Luebeck, Germany; 2 Department of Molecular Genetics, Erasmus MC Cancer Institute, Erasmus University Medical Centre Rotterdam, 3015 GD Rotterdam, The Netherlands; i.chaves@erasmusmc.nl

Among the many factors affecting general health and resilience to disease, lifestyle is at the same time the most controllable and the most influential factor [1,2,3]. Non-communicable diseases account for more than 60% of deaths worldwide. Consequently, the continuous rise in chronic disease cases is the most pressing challenge to global health [4]. In most industrial countries, chronic disorders have become the main cause of poor health, disabilities, and premature death, consequently accounting for a dominant fraction of expenditures in the healthcare system [5]. The burden of chronic disease in Western countries such as the United States is rooted in three key factors: a high prevalence of risky lifestyles and behaviors [6,7], social and environmental conditions that have an adverse impact on health state, and an increased life expectancy, which results in more older people suffering from one or more chronic conditions [8].

A healthy diet, moderate and regular exercise, and sufficient amounts of high-quality sleep form the basis of a healthy lifestyle. Healthy diet choices and regular physical exercise can dramatically delay or prevent the incidence of chronic diseases [9,10]. Sleep is another important health-promoting factor that is still neglected in modern societies [11,12,13]. People's average sleep times continue to decrease, while the prevalence of sleep disorders is on the rise [14]. Lasting adoption of healthy habits and behaviors can effectively prevent or control chronic diseases. Time of day is an important—albeit too often disregarded—factor when studying the health effects of diet, sleep, and exercise. Taking timing into account for lifestyle interventions might lead to additional health benefits and at the same time improve compliance [15,16]. The studies in this Special Issue shed new light on behavioral rhythms and their effect on metabolic disorders and cancer.

With disease progression, most cancer cells develop specific metabolic profiles, making them highly sensitive to changes in nutrient supplies and general metabolic state. These effects have brought diet interventions to the attention of oncologists and cancer researchers. The risk of colorectal cancer (CRC), for example, is markedly influenced by dietary patterns and the composition of the gut microbiome. However, so far, the interaction between these two factors has remained unclear. The study by Cai et al. (contribution 1) examined this relationship, hypothesizing that different types of dietary nutrient composition may differentially affect colorectal cancer risk in individuals, dependent on gut microbiome composition. This case–control study involving 410 Han Chinese individuals compared two dietary patterns and three gut microbiota enterotypes to classify 250 colorectal neoplasm cases. It found that a healthy diet based on vegetables, fruits, and dairy products lowers the CRC risk in subjects with type-I (dominated by *Bacteroides* and *Lachnoclostridium*) and type-II gut microbiota enterotypes, at an adjusted odds ratio of 0.66.

Diet composition and microbiome regulation similarly affect specific metabolic diseases such as metabolic-associated steatotic liver disease (MASLD, formerly NAFLD), which is currently one of the most prevalent metabolic disorders worldwide. In turn, lifestyle interventions remain the most effective treatment for MASLD and its sequelae such as metabolic-associated steato-hepatitis (MASH) and liver cirrhosis to date. Bianco et al. (contribution 2) aimed at estimating the effect of a Mediterranean diet and exercise over a one-year period on the longitudinal trajectories of glucose metabolism in MASLD patients. There was an early onset and steady decline of HbA1c levels in participants with moderate and severe steatosis, while this effect became apparent only after nine months in patients with early-stage MASLD. Effects were persistent over a one-year period. Gu et al. (contribution 3), on the other hand, studied the association of 24-hour behavioral rhythms with MASLD in a cohort of 4502 overweight/obese adults. Comparing the lowest quintile to the highest, participants with higher activity amplitudes had a reduced risk of MASLD. Similarly, individuals in the highest quintile for fasting duration and feeding rhythm score had a decreased MASLD risk. These associations were more pronounced among individuals with obesity, underscoring the potential of behavioral interventions for MASLD therapy.

Social jetlag (SJL) describes a discrepancy between sleep hours on week-/workdays compared to weekends/free days [17]. SJL has been linked to decreased metabolic health. Nitta et al. (contribution 4) compared meal patterns in a group of Japanese participants aged 20–59 years over one month using a food logging mobile app. In participants with an early (morning) chronotype and little SJL, a higher daily intake was seen for fibers, potassium, phosphorus, magnesium, and vitamin K. On the other hand, dinner energy and nutrient intake of carbohydrates, proteins, and total lipids, as well as sodium and saturated fatty acids, were lower in this group. Utilization of app data may help to establish references for dietary intake and informed eating patterns over the 24-hour day.

In many Asian countries, eating patterns have adapted to a “Westernized lifestyle” over the last decades, while at the same time, the prevalence of type 2 diabetes (T2DM) has markedly increased. Yang et al. (contribution 5) investigated the hypothesis that healthy eating habits are associated with reduced T2DM risk in adults with sex-specific differences. Their data show that T2DM was more frequently observed in older men of lower education state with lower income. In addition, T2DM patients were more often married and lived in rural environments compared to non-T2DM subjects. Blood data showed that non-diabetic women had higher intake levels of vitamin C, calcium, fatty acids, retinol, and vitamin B2 compared to T2DM subjects, while this effect was not seen in males. In women, but not in men, healthy eating scores were inversely associated with T2DM. These data suggest sex-specific effects of diet habits on T2DM development.

A potent and increasingly recognized factor controlling appetite and food choice is sleep. Reduced sleep duration increases hunger, appetite, and food intake [18]. Gangitano et al. [contribution 6] summarize the current evidence on and mechanisms of this interaction, combining findings from animal models and human studies. They conclude that deciphering the molecular regulatory pathways of sleep and its physiological outputs will have a major impact on ameliorating metabolic health in vulnerable individuals. Vice versa, weight loss therapies have strong benefits for cardiometabolic health and the quality of sleep.

In a related study, Meyhöfer et al. (contribution 7) describe the impact of sleep phase timing, as opposed to its duration, on the regulation of parameters of appetite and hunger. In a small-scale laboratory setup (n = 15), participants were tested for neuroendocrine factors and appetite ratings after 4-hour sleep intervals during the early or late night. Blood ghrelin levels, hunger and appetite rates, and the drive for food consumption were elevated after sleep loss during the late but not the early part of the night. Leptin levels, on the other hand, were not affected by sleep timing. These data emphasize the metabolic relevance of circadian sleep regulation.

To summarize, behavioral interventions promote metabolic health through lifestyle changes, including diet improvements, increased physical activity, stress management, and sustainable behavioral changes [19]. These interventions help individuals maintain a healthy metabolic state with high insulin sensitivity and low vulnerability for metabolic conditions like obesity and T2DM. This Special Issue highlights the role of timing in this context. Discriminating parameters such as chronotype are assessed and strategies are devised to implement chronomedical approaches in lifestyle therapies.

## References

[1] Swaby A., Atallah A., Varol O., Cristea A., Quail D.F. (2023). Lifestyle and Host Determinants of Antitumor Immunity and Cancer Health Disparities. Trends Cancer.

[2] Younossi Z.M., Zelber-Sagi S., Henry L., Gerber L.H. (2023). Lifestyle Interventions in Nonalcoholic Fatty Liver Disease. Nat. Rev. Gastroenterol. Hepatol..

[3] Khan T.A., Field D., Chen V., Ahmad S., Mejia S.B., Kahleová H., Rahelić D., Salas-Salvadó J., Leiter L.A., Uusitupa M. (2023). Combination of Multiple Low-Risk Lifestyle Behaviors and Incident Type 2 Diabetes: A Systematic Review and Dose-Response Meta-Analysis of Prospective Cohort Studies. Diabetes Care.

[4] WHO (2022). Non-Communicable Diseases Progress Monitor 2022.

[5] Bauer U.E., Briss P.A., Goodman R.A., Bowman B.A. (2014). Prevention of Chronic Disease in the 21st Century: Elimination of the Leading Preventable Causes of Premature Death and Disability in the USA. Lancet.

[6] Xu F., Mawokomatanda T., Flegel D., Pierannunzi C., Garvin W., Chowdhury P., Salandy S., Crawford C., Town M., Centers for Disease Control and Prevention (CDC) (2014). Surveillance for Certain Health Behaviors among States and Selected Local Areas—Behavioral Risk Factor Surveillance System, United States, 2011. MMWR Surveill. Summ..

[7] National Center for Health Statistics (US) (2013). Health, United States, 2012: With Special Feature on Emergency Care.

[8] Centers for Medicare and Medicaid Services (2012). Chronic Conditions among Medicare Beneficiaries, 2012 ed..

[9] Isath A., Koziol K.J., Martinez M.W., Garber C.E., Martinez M.N., Emery M.S., Baggish A.L., Naidu S.S., Lavie C.J., Arena R. (2023). Exercise and Cardiovascular Health: A State-of-the-Art Review. Prog. Cardiovasc. Dis..

[10] Volpp K.G., Berkowitz S.A., Sharma S.V., Anderson C.A.M., Brewer L.C., Elkind M.S.V., Gardner C.D., Gervis J.E., Harrington R.A., Herrero M. (2023). Food Is Medicine: A Presidential Advisory From the American Heart Association. Circulation.

[11] Schmid S.M., Hallschmid M., Schultes B. (2015). The Metabolic Burden of Sleep Loss. Lancet Diabetes Endocrinol..

[12] Li X., Huang D., Liu F., Li X., Lv J., Wu Q., Zhao Y. (2022). Sleep Characteristics and Cancer-Related Outcomes: An Umbrella Review of Systematic Reviews and Meta-Analyses of Observational Studies. J. Clin. Med..

[13] Hany M., Abouelnasr A.A., Abdelkhalek M.H., Ibrahim M., Aboelsoud M.R., Hozien A.I., Torensma B. (2023). Effects of Obstructive Sleep Apnea on Non-Alcoholic Fatty Liver Disease in Patients with Obesity: A Systematic Review. Int. J. Obes..

[14] Gomes S., Ramalhete C., Ferreira I., Bicho M., Valente A. (2023). Sleep Patterns, Eating Behavior and the Risk of Noncommunicable Diseases. Nutrients.

[15] Kramer A., Lange T., Spies C., Finger A.-M., Berg D., Oster H. (2022). Foundations of Circadian Medicine. PLoS Biol..

[16] Cederroth C.R., Albrecht U., Bass J., Brown S.A., Dyhrfjeld-Johnsen J., Gachon F., Green C.B., Hastings M.H., Helfrich-Förster C., Hogenesch J.B. (2019). Medicine in the Fourth Dimension. Cell Metab..

[17] Roenneberg T., Kuehnle T., Pramstaller P.P., Ricken J., Havel M., Guth A., Merrow M. (2004). A Marker for the End of Adolescence. Curr. Biol..

[18] Rogers E.M., Banks N.F., Jenkins N.D.M. (2023). The Effects of Sleep Disruption on Metabolism, Hunger, and Satiety, and the Influence of Psychosocial Stress and Exercise: A Narrative Review. Diabetes Metab. Res. Rev..

[19] Zucatti K.P., Teixeira P.P., Wayerbacher L.F., Piccoli G.F., Correia P.E., Fonseca N.K.O., Moresco K.S., Guerra B.A., Maduré M.G., Farenzena L.P. (2022). Long-Term Effect of Lifestyle Interventions on the Cardiovascular and All-Cause Mortality of Subjects With Prediabetes and Type 2 Diabetes: A Systematic Review and Meta-Analysis. Diabetes Care.

